# Apical Localisation of Crumbs in the Boundary Cells of the *Drosophila* Hindgut Is Independent of Its Canonical Interaction Partner Stardust

**DOI:** 10.1371/journal.pone.0094038

**Published:** 2014-04-07

**Authors:** Alexandra Kumichel, Elisabeth Knust

**Affiliations:** Max-Planck-Institute of Molecular Cell Biology and Genetics, Dresden, Germany; Texas A&M International University, United States of America

## Abstract

The transmembrane protein Crumbs/Crb is a key regulator of apico-basal epithelial cell polarity, both in *Drosophila* and in vertebrates. In most cases studied so far, the apical localisation of *Drosophila* Crumbs depends on the interaction of its C-terminal amino acids with the scaffolding protein Stardust. Consequently, embryos lacking either Crumbs or Stardust develop a very similar phenotype, characterised by the loss of epithelial tissue integrity and cell polarity in many epithelia. An exception is the hindgut, which is not affected by the loss of either gene. The hindgut is a single layered epithelial tube composed of two cell populations, the boundary cells and the principal cells. Here we show that Crumbs localisation in the principal cells depends on Stardust, similarly to other embryonic epithelia. In contrast, localisation of Crumbs in the boundary cells does not require Stardust and is independent of its PDZ domain- and FERM-domain binding motifs. In line with this, the considerable upregulation of Crumbs in boundary cells is not followed by a corresponding upregulation of its canonical binding partners. Our data are the first to suggest a mechanism controlling apical Crumbs localisation, which is independent of its conserved FERM- and PDZ-domain binding motifs.

## Introduction

A hallmark of epithelial cell polarity is the separation of the plasma membrane into an apical side facing the outside or a lumen, and a baso-lateral side, which makes contact with the neighbouring cells and/or the basal membrane. The *zonula adherens* (ZA), an adhesion belt surrounding the apex of epithelial cells, marks the boundaries between them. The apico-basal subdivision of the plasma membrane becomes manifest by the uneven distribution of various proteins, many of which serve membrane domain-specific functions. Proper targeting of proteins to and their maintenance in the respective membrane is of utmost importance for epithelial development and homeostasis. Mechanisms controlling these processes include exo- and endocytosis, protein-protein and protein-lipid interactions to stabilise proteins in the membrane, or recycling and degradation of proteins. In addition, the synthesis of the right amounts of membrane-specific proteins, their modifications and proper targeting are important regulators of apico-basal polarity [reviewed in [1], [2], [3], [4], [5]].

One of the key regulators of epithelial polarity in the *Drosophila* embryo is the Crumbs protein complex, the core components of which are the transmembrane protein Crumbs (Crb) and the scaffolding proteins Stardust (Sdt), *D*Lin-7 and *D*PATJ. Other components, such as *D*Par6, a member of the Par protein group, or Yurt, a negative regulator of Crb, can be transiently recruited into the complex [reviewed in [6], [7]]. *crb* and *sdt* mutant embryos are unable to maintain apico-basal polarity in many of their epithelia. This eventually results in a complete breakdown of tissue integrity due to a failure to position and maintain the ZA, followed by apoptosis in some tissues, e.g. the epidermis [8], [9], [10], [11]. Similar defects in epithelial integrity are observed in mice lacking Crb2 or Crb3 [12], [13]. Conversely, overexpression of *Drosophila* Crb can lead to an expansion of the apical membrane domain, both in embryonic epithelial cells [14] and in photoreceptor cells [15], [16], [17]. These results suggest that the amount of Crb has to be tightly regulated in order to maintain the proper size and differentiation of the apical membrane.

So far, little is known about the mechanisms that ensure the proper levels of Crb and other members of the complex at the apical membrane and hence the balance between apical and baso-lateral membrane domains. Exo84, a component of the exocyst, and the retromer, which controls recycling of Crb, as well as Cdc42 and Rab11 are essential for localising and maintaining Crb on the apical surface [18], [19], [20], [21], [22]. In most epithelial tissues of the *Drosophila* embryo a direct interaction between the C-terminal ERLI motif of the short cytoplasmic tail of Crb and the PDZ (**P**SD-95/**D**iscs-large/**Z**O-1)-domain of Sdt is essential for the localisation of both proteins in the subapical region (SAR), a portion of the apical plasma membrane just apical to the ZA. Loss of either *crb* or *sdt* results in the loss of the respective other protein from the apical membrane and thus to a very similar mutant embryonic phenotype [9], [23], [24].

Strikingly, the *Drosophila* embryonic hindgut does not show any obvious defect in polarity or morphogenesis in *crb* or *sdt* mutant embryos, although it expresses the Crb complex from early on. The hindgut is a single layered epithelial tube, which is subdivided – from anterior to posterior - into the small intestine, the large intestine and the rectum [reviewed in [25]]. The large intestine is additionally patterned along the dorso-ventral axis, with the dorsal and ventral compartments separated by a single row of epithelial cells, called the boundary cells (BCs). These three compartments can be distinguished by the morphology of their cells and different gene expression patterns, but their specific functions later on are only partially understood. While the *engrailed*-expressing dorsal cells become specialized for water and ion absorption [26], a specific function of the *engrailed*-negative, *Delta*-expressing ventral cells has not yet been described. The BCs not only express several transcription factors distinct from those expressed in the dorsal and ventral compartment, but also exhibit much higher levels of Crb on their apical surface in comparison to the neighbouring, principal cells (PCs) [27], [28], [29]. In addition, BCs are more elongated than PCs and develop more pronounced apical microvilli [29]. Hence, the *Drosophila* large intestine provides an ideal system not only to study pattern formation, but also to unravel the requirement for cell-type specific differentiation and morphogenesis of epithelial cells in a single epithelia tube. In particular, the previously demonstrated link between Crb abundance and apical differentiation motivated us to study in more detail the requirement of this polarity regulator for BC differentiation. Here we show that BCs use a so far not described, Sdt-independent mechanism to accumulate Crb on the apical surface.

## Materials and Methods

Flies were kept at 25°C. The following stocks/mutant alleles were used: OregonR as wild-type control, *crb^11A22^* [30], *crb^GX24^* [31], *crb^8F105^* [30], [32] *foscrb_Y10A,ΔERLI_*; *crb^GX24^* [33], *UAS-crb^30.12e^* [14] called *UAS-crb^full^* here, *en-*Gal4 [34]. Mutant stocks were balanced over *TM3, Twist-Gal4, UAS-EGFP* (Bloomington Stock Center).

### Immunohistochemistry

Embryo collection, fixation and antibody staining were conducted as previously described [33]. The following primary antibodies were used: rabbit anti-Baz (1∶100) [35], rat anti-Crb 2.8 (1∶1000) [36], rabbit anti-Crb intra (1∶400 raised against the peptide NKRATRGTYSPSAQE; unpublished), rabbit anti-*D*PATJ (1∶1000) [36], mouse anti-Dlg 4F3 (1∶400; Developmental Studies Hybridoma Bank [DSHB]), rabbit anti-Lgl (1∶100) [37], rabbit anti-*D*Lin-7 (1∶100) [38], rat anti-*D*Par6 (1∶500; kindly provided by A. Wodarz), rabbit anti-PKCζ C20 (1∶400; Santa Cruz Biotechnology), rabbit anti-Sdt-PDZ (1∶500) [39], rabbit anti-Sas (1∶500; kindly provided by E. Organ and D. Cavener), rabbit anti-Scrib (kindly provided by D. Bilder), mouse anti-α-Spectrin SA9 (1∶400; DSHB). Secondary antibodies used in this study were conjugated to Alexa Flour 488, −568 and −647 (Life Technologies). Stained embryos were mounted in glycerine propyl gallate (75% glycerol, 50 mg/ml propyl gallate).

Cryosections were prepared from fixed and stained embryos. For cryopreservation specimens were first incubated in 10% sucrose for 30 min at room temperature, and then in 25% sucrose over night at 4°C. Embryos were embedded in tissue-freezing medium (NEG50, Thermo Scientific), frozen on dry ice and stored at −80°C. Cryosections (10 μm) were made with a Microm Cryo-Star HM560M, collected on coated glass slides (Marienfeld) and mounted in DABCO-containing (Sigma) Mowiol (Calbiochem).

Images were taken with a LSM Zeiss 510 using a Zeiss Plan-Achromat 63x lens. All quantifications were performed using Fiji software [40]. To measure the fluorescence intensity of Crb in BCs and PCs a region of interest (ROI) was defined around the respective cell as well as in an area without fluorescent objects, which was used for background subtraction. Whole cell signal corrected per area was calculated using the following formula: (whole cell signal – area of selected cell x mean fluorescence of background readings) / area of selected cell. For the analysis of each cell type 18 regions of six individual hindguts were selected. The statistical significance was assessed by two-sided Student's *t-*test in Microsoft Excel. Colocalization analyses were performed using the JACOP plugin of the Fiji software [41].

For colocalization analysis in the BCs a section was used that showed a lateral view of the hindgut. The ROI was drawn on the apical surface of individual BCs. For colocalization analysis in PCs a section was used that showed a longitudinal section through the hindgut tube. The ROI was drawn on the SAR of the PCs. For the analysis of each cell type 12 regions of four individual hindguts were selected. Box graphs and statistical analysis were performed using Microsoft Excel. For image processing and analysis Fiji and Adobe Photoshop CS5 were used and Adobe Illustrator CS5 for image assembly.

### Transmission Electron Microscopy

Sections were prepared as described in [42] with modifications. In brief, fixation of devitellinized embryos in 0.1 M phosphate buffer (pH 7.2) was performed in 2.5% glutaraldehyde, followed by fixation in 1% osmium tetroxide/2% glutaraldehyde, followed by 2% OsO_4_. After dehydration embryos were embedded in Araldite. Semithin sections (2.5 μm) and ultra thin sections (70 nm) were prepared with the Leica Ultracut UCT microtome. Ultrathin sections were contrasted and analysed with a FEI Tecnai 12 Bio Twin. Microvilli length was measured using Fiji software and statistical analysis was performed using Microsoft Excel. For image processing and analysis Fiji software and Adobe Photoshop CS5 were used and Adobe Illustrator CS5 for image assembly. The statistical significance was assessed by two-sided Student's *t-*test in Microsoft Excel.

## Results

### Crb, but not other members of the Crb complex is upregulated in the BCs

**Figure 1:**
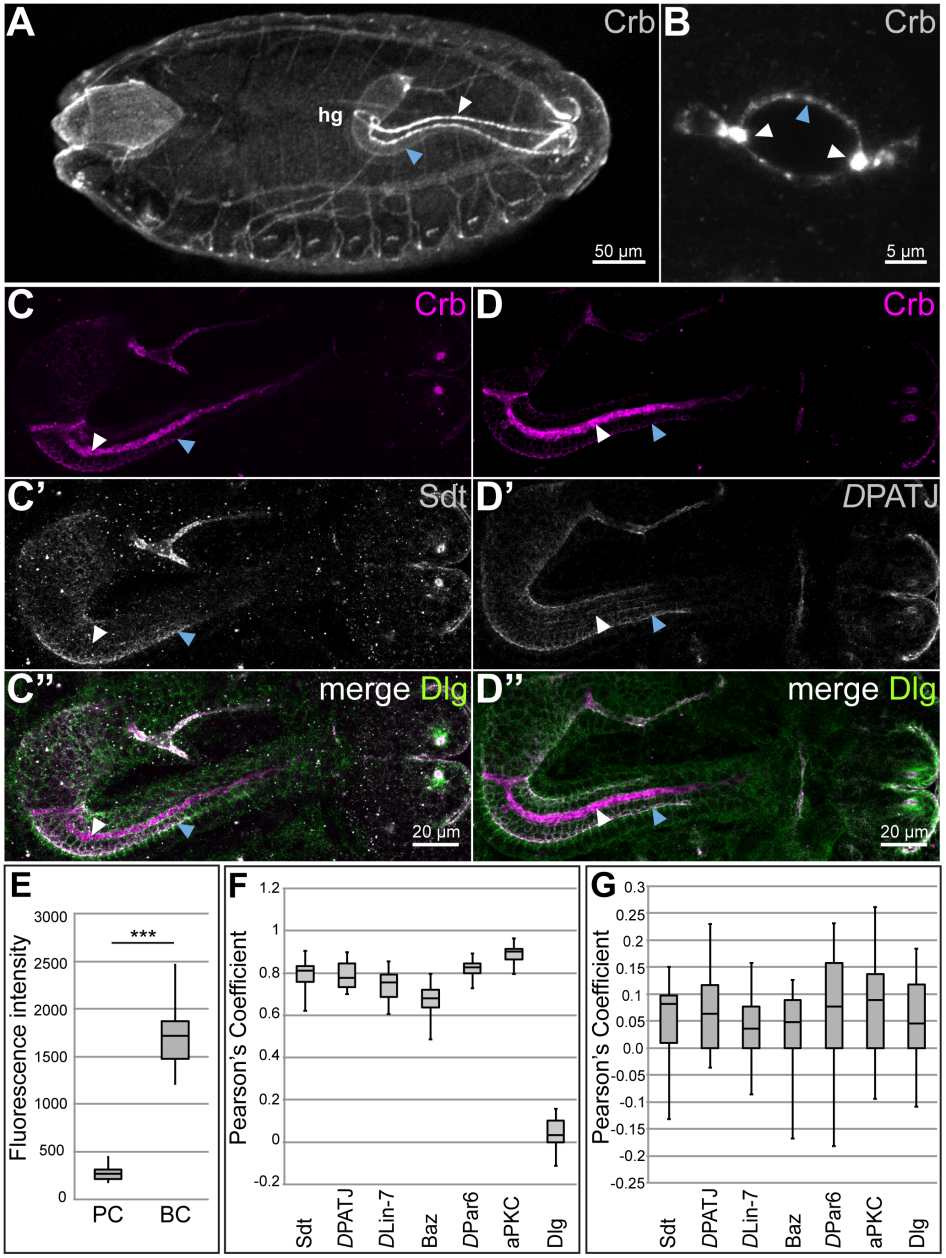
Crb, but not the Crb complex members Sdt and *D*PATJ, is enriched in the BCs of the embryonic hindgut. (A–D″) Confocal microscope images of stage 15 wild-type embryos. (**A**) Dorsal view of a whole mount embryo stained with anti-Crb, showing apical localisation in the PCs (blue arrowhead) and strong enrichment in the BCs (white arrowhead) of the hindgut (hg). Anterior is left. (**B**) Cross section through the large intestine stained with anti-Crb to show strong apical accumulation of Crb in the BCs (white arrowheads) and SAR localisation in the PCs (blue arrowhead). (**C–D″**) Confocal microscope images of embryonic hindguts stained with anti-Crb (magenta in C, C″, D and D″), anti-Sdt (grey in C′ and C″) and anti-*D*PATJ (grey in D′ and D″) as well as anti-Dlg (green in C″ and D″). BCs (white arrowheads) accumulate Crb (C, C″, D, D″) but not Sdt (C′ and C″) or *D*PATJ (D′, and D″). PCs (C–D″, blue arrowheads) localise Crb (C, C″, D, D″), Sdt (C′ and C″) and *D*PATJ (D′ and D″) in the SAR. (**E**) Box Plot showing the fluorescence intensity of anti-Crb staining in PCs and BCs of stage 15 wild-type embryos. The line within the box represents the median value; the whiskers represent the maximum and minimum values; *** indicate p-value <0.001, assessed by two-sided Student's *t-*test.(**F, G**) Box Plot showing the Pearson's correlation coefficient of Crb and Sdt, *D*PATJ, *D*Lin-7, Baz, *D*Par6, aPKC and Dlg in PCs (F) and BCs (G) of stage 15 wild-type embryos. The line within the box represents the median value; the whiskers represent the maximum and minimum values. Note the difference in the scale of the Pearson's correlation coefficient in F and G.

*crb* RNA and protein have been shown to be strongly upregulated in BCs of the *Drosophila* embryonic hindgut [27], [28], [29]. Crb protein is spread across the apical pole of the BCs in stage 15 (Fig. 1A, B, white arrowheads) and older (stage 16) embryos [33]. In contrast, the majority of the hindgut cells, which we will call principle cells (PCs) from now on, express low levels of Crb, which is restricted to the subapical region (SAR), apical to the *zonula adherens* (ZA), as in most other embryonic epithelia (Fig. 1A, B, blue arrowheads). Crb is about 6.5 fold more abundant in BCs than in PCs (Fig. 1E).

The short cytoplasmic domain of Crb is required to localise other members of the Crb complex to the SAR by direct interaction between the C-terminal ERLI motif of Crb and the PDZ domain of Sdt. Consequently, in many epithelia loss of *crb* results in loss of the scaffolding core components of the Crb complex, Sdt, *D*PATJ and *D*Lin-7. Furthermore, overexpression of the membrane bound intracellular domain of Crb can recruit other Crb complex members to ectopic sites, but only in the presence of an intact PDZ-domain binding motif [17], [43]. Therefore we asked, whether upregulated Crb in the BCs also results in the upregulation of Sdt, *D*PATJ and *D*Lin-7. Unlike Crb, none of the three proteins is upregulated in the BCs, but all show a similar level of expression as in PCs (Fig. 1C′, C″ and 1D′, D″ and data not shown) and co-localise with Crb, as demonstrated by determining the Pearson's Correlation Coefficient (Fig. 1F). None of them is spread on the apical pole of the BCs, but all are restricted to the SAR. This result is in contrast to data published previously (although not shown) [27], arguing that *D*PATJ (in this paper still called Discs Lost, Dlt) is upregulated in the BCs. From our data we conclude that, unlike in most epithelial cells, in which the regulation of the amount of Crb and Sdt seem to be tightly coupled [23], [24], [44], the increased level of Crb in the BCs is not associated with a corresponding increase of other core components of the complex.

### Loca lisation of other polarity regulators is not altered in the BCs

**Figure 2:**
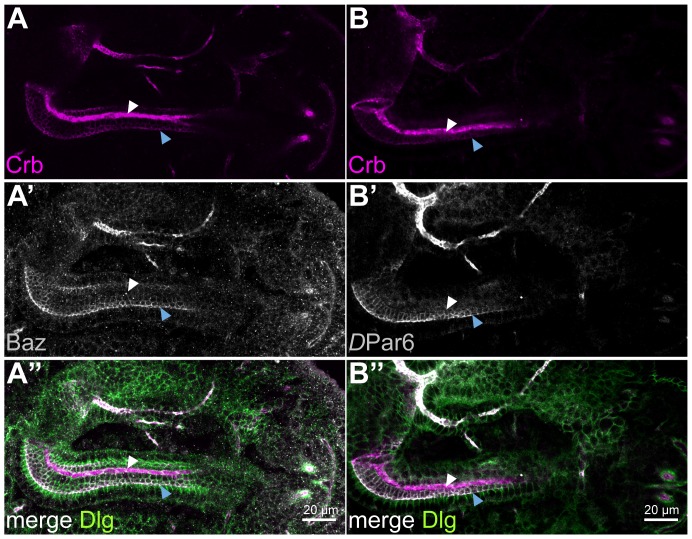
Localisation of apical and baso-lateral polarity proteins is not altered in BCs. Confocal microscope images of stage 15 wild-type embryonic hindguts (BCs: white arrowheads, PCs: blue arrowheads). Anterior is left. (**A–A″**) Hindgut stained for the polarity markers Crb (magenta), Baz (grey) and Dlg (green). Only Crb is upregulated in the BCs (A and A″), while Baz (A′ and A″) and Dlg (A″) show the same amount and localisation as in the PCs. (**B–B″**) Hindgut stained for the polarity markers Crb (magenta) and *D*Par6 (green). Crb is enriched in the BCs (B and B″) but *D*Par6 localises only to the SAR as in the PCs (B′ and B″).

Beside the Crb complex, other proteins and protein complexes are required for the establishment and maintenance of apico-basal polarity in epithelial cells. Amongst these are the scaffolding proteins Bazooka (Baz), the *Drosophila* orthologue of Par3, *D*Par6 and the atypical protein kinase C (aPKC). Baz often, but not always, forms a complex with the *D*Par6/aPKC heterodimer at the SAR, occasionally overlapping with the ZA [45], [46], [47], [48]. Several results suggest a close connection between members of the Crb and the Baz/*D*Par6/aPKC complexes [see [7], [49] for recent reviews]. For example, the single PDZ-domain of *D*Par6/Par6 can directly bind to the C-terminus of Crb/CRB3 [50], [51]. In the BCs of the hindgut, however, neither *D*Par6 nor Baz or aPKC are upregulated. All three are restricted to the SAR as in PCs (Fig. 2A′-A″, B′-B″ and data not shown), where they co-localise with Crb (Fig. 1F).

Members of the conserved Scribble (Scrib) module, including the multi PDZ-domain protein Scrib, the membrane-associated guanylate kinase (MAGUK) Discs large (Dlg) and the WD40-domain protein Lethal giant larvae (Lgl), localise at the baso-lateral membrane of many epithelial cells and antagonise the function of apical regulators [52], [53], [54] [see [55], [56] for recent reviews]. In the hindgut, all three proteins are localised at the baso-lateral membrane, both in BCs and PCs (Fig. 2A″ and data not shown). To summarise, only the transmembrane protein Crb, but none of the other known polarity regulators tested, is upregulated in the BCs of the embryonic hindgut.

### Crb stabilisation in the apical membrane domain of the BCs is independent of Sdt

**Figure 3:**
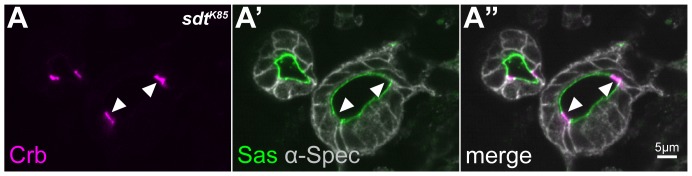
Apical localisation of Crb in BCs is independent of its known interaction partner Sdt. Confocal microscopy images of a cross section through the large intestine of a stage 15 homozygous *sdt^K85^* mutant embryo stained with anti-Crb (magenta), anti-α-Spectrin (grey) and anti-Sas (green). Crb is upregulated and apically localised in the BCs (white arrowheads), but is not detectable in the SAR of the PCs (A, A″). Sas, an apical marker of the monolayered epithelial tube, is reduced in the BCs (A′, A″).

In most embryonic epithelial cells Crb and Sdt are strongly dependent on each other with respect to their amount, localisation and stability [23], [24], [44]. As a consequence, loss of either *crb* or *sdt* results in the same mutant phenotype [9], [30], [57]. Given the observation that the upregulation of Crb is not reflected by an upregulation of Sdt in the BCs of the hindgut, we asked whether stabilization of Crb in these cells depends on Sdt at all. Therefore, we studied localization of Crb in embryos mutant for *sdt^K85^*, a complete loss of function allele [39]. In the BCs of homozygous *sdt^K85^* embryos, Crb protein is still apically localised and strongly upregulated. In contrast, Crb is not detectable in the SAR in the PCs of the hindgut, as has been described for most epithelia (Fig. 3A–A″; compare with Fig. 1D). The hindgut tube remains single layered, as visualized by α-Spectrin staining, and cells maintain proper apico-basal polarity, as shown by apical localisation of Stranded-at-Second (Sas), a marker for the apical membrane [14] (Fig. 3). In conclusion, Sdt is not required for apico-basal polarity of the hindgut epithelium, or for the stabilisation of Crb in the apical membrane of the BCs.

### Crb stabilisation in the apical membrane domain is independent of interactions via its PDZ- and FERM-domain binding motifs

**Figure 4:**
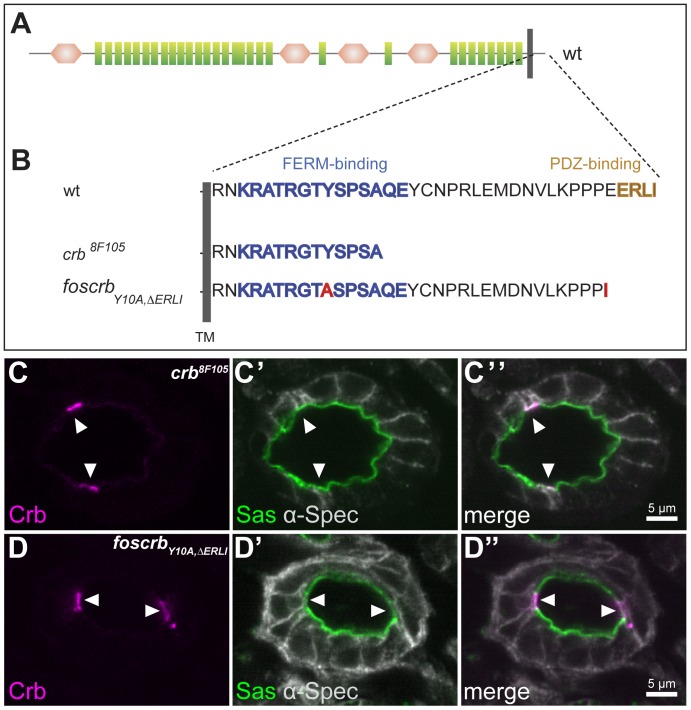
Localisation of Crb in BCs is independent from its known protein binding motifs. (**A**) Schematic representation of the Crb protein and its variants used in this study. Green rectangles: EGF-like repeats, brown hexagons: laminin A G like domains, grey bar: transmembrane domain (TM). (**B**) Amino acid sequences of the cytoplasmic tails of wild-type and mutant Crb proteins used in this study. Blue: FERM domain-binding motif, brown: PDZ domain-binding motif. Red: point mutations. (**C–D″**) Confocal microscopy images of cross sections through the large intestine of stage 15 homozygous *crb^8F105^* (C–C″) and *foscrb_Y10A,ΔERLI_* (D–D″) embryos stained with anti-Crb (magenta), anti-α-Spectrin (grey) and anti-Sas (green). Crb is upregulated and apically localised in the BCs (white arrowheads), but is not detectable in the PCs.

The cytoplasmic domain of Crb contains two protein-protein interaction domains, a C-terminal PDZ-domain binding motif, -ERLI, and a FERM (protein **4**.1/**e**zrin/**r**adixin/**m**oesin)-domain binding motif [43], [58]. Given the observation that the apical enrichment of Crb in the BCs is independent of Sdt, we were interested to know whether another protein containing a PDZ-domain could be involved in Crb stabilisation in these cells. To address this question, we studied Crb localisation in the allele *crb^8F105^*. A point mutation in this allele induces a premature stop codon, resulting in the synthesis of a truncated Crb protein that lacks the last 23 amino acids of the intracellular domain, including the PDZ-domain binding motif (Fig. 4B). Yet, the phenotype of homozygous *crb^8F105^* mutant embryos resembles that of complete loss of function alleles [32]. The truncated Crb protein produced in homozygous mutant *crb^8F105^* embryos is not apically localised in the majority of epithelial cells [32], including the PCs (Fig. 4C–C″). In contrast, the truncated protein is still enriched in the apical pole of the BCs (Fig. 4C–C″, white arrowheads).

The second well-established protein-protein interaction domain of the cytoplasmic tail of Crb is a conserved FERM-domain binding motif. In *Drosophila*, two binding partners have been identified so far, the FERM proteins Yurt and Expanded [58], [59]. To address whether the accumulated Crb protein in the BCs is stabilised via interactions through its FERM-domain binding site, we studied the localisation of Crb in embryos lacking endogenous *crb*, but containing a transgene encoding a Crb protein with mutations in the FERM-domain binding and PDZ-domain binding motifs, called *foscrb_Y10A,ΔERLI_*. The Crb protein encoded by this transgene carries an exchange of a conserved tyrosine residue in the FERM-domain binding motif by an alanine (Y10A). In addition, the PDZ-domain binding motif is removed (ΔERLI) (Fig. 4B). This mutant protein is unable to rescue any defect of *crb* mutant embryos, and the phenotype of *foscrb_Y10A,ΔERLI_*; *crb* mutant embryos resembles that of embryos with no functional *crb* gene, in that epithelial integrity is lost in most embryonic tissues [33]. In the hindgut, the mutant Crb protein is accumulated and stabilised in the apical membrane domain of the BCs (Fig. 4D–D″), while no localised signal was detected in the PCs. As in other *crb* alleles, the hindgut maintains its monolayered tubular structure and proper apico-basal polarity in *crb^8F105^* and *foscrb_Y10A,ΔERLI_*; *crb* mutant embryos, as revealed by proper apical localisation of Sas (Fig. 4C′, C″, D′, D″, green staining).

From these results we conclude, that, unlike in most embryonic epithelia, the PDZ-domain binding motif of Crb is not required for its stabilisation and enrichment on the apical membrane of the BCs. This observation excludes Sdt, *D*Par6 and other PDZ-domain containing proteins as candidates for its stabilisation. Similarly, apical accumulation of Crb in BCs does not depend on an intact FERM-domain binding motif. These results suggest that the BCs use a different way to stabilise Crb on the apical surface, which is independent of its known interactors.

### Loss of *crb* in BCs affects the length of microvilli

**Figure 5:**
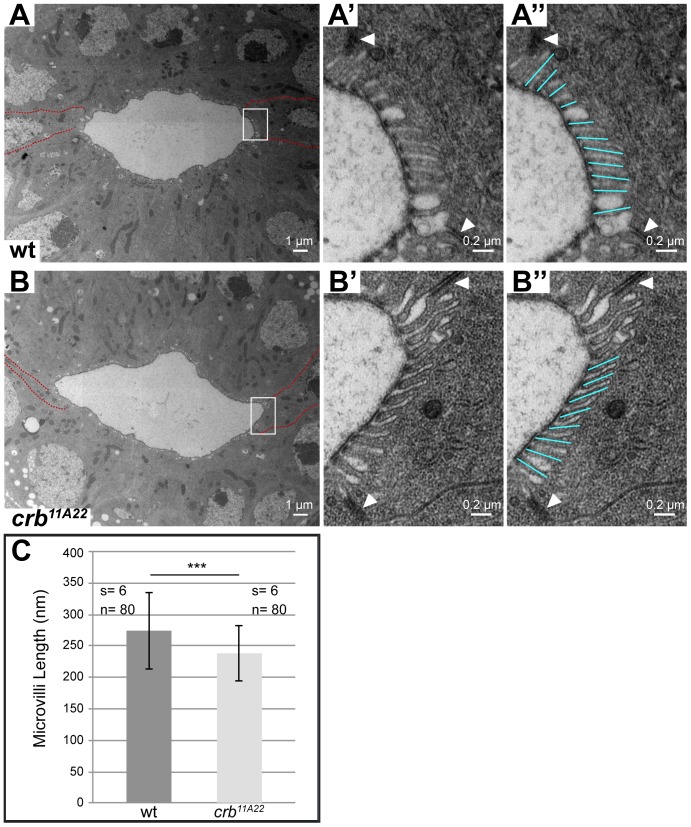
Loss of Crb from the BCs alters the apical membrane structure. (**A–B″**) Electron micrographs of cross sections through the large intestines of stage 16 wild-type (A–A″) and homozygous mutant *crb^11A22^* (B–B″) embryos. In A and B, BCs are outlined by red lines, the rectangles indicate areas enlarged in A′, A″, B′ and B″. White arrowheads in A′, A″, B′ and B″ point to the adherens junctions between the BCs and PCs. BCs form longer and more regular microvilli than the PCs in wild-type (A–A″) and homozygous *crb^11A22^* mutant embryos (B–B″). (**C**) Graph showing the mean length of microvilli in the BCs of stage 16 wild-type and *crb^11A22^* mutant embryos ± standard deviation. s refers to the number of embryos analysed; n refers to the number of microvilli analysed. ***indicate p-value <0.001, assessed by two-sided Student's *t-*test.

While loss of Crb results in a reduction of the apical membrane in some cells, its overexpression can induce an expansion of the apical membrane. Therefore, we asked whether the high level of Crb in the BCs is responsible for the increased length of microvilli observed in these cells. Increase in microvilli length becomes obvious from stage 14 onwards [26], [27], [60]. In fact, the length of the microvilli in the BCs of stage 16 *crb^11A22^* mutant embryos is slightly, but significantly reduced compared to that in wild-type BCs (Fig. 5).

**Figure 6:**
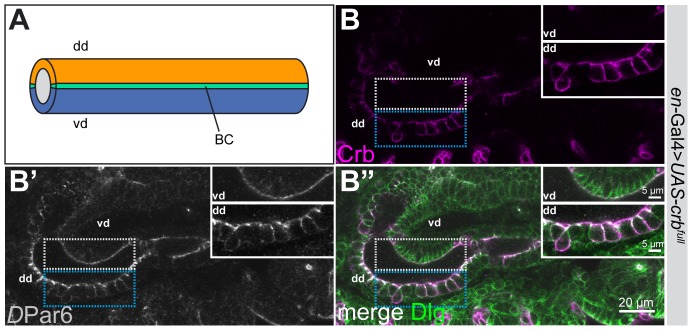
Crb overexpression in PCs leads to an expansion of the apical membrane domain. (**A**) Schematic representation of the large intestine with the dorsal domain (dd) in orange, the ventral domain (vd) in blue and the BCs in green. (**B–B″**) Confocal microscope images of a stage 15 embryonic hindgut expressing *UAS-crb^full^* under the control of *en-*GAL4 in the PCs of the dorsal domain. The hindgut is stained with anti-Crb (magenta), anti-*D*Par6 (grey) and anti-Dlg (green). The insets show higher magnifications of the vd (outlined by the grey dotted line), which serves as control tissue and the dd (outlined by the blue dotted line) where the altered apico-basal polarity in cells overexpressing Crb is highlighted (due to the very strong overexpression of Crb in the dd, the gain of the microscope was strongly reduced).

To find out whether overexpression of Crb has an effect on the differentiation of the apical surface of the PCs, we overexpressed full-length Crb protein in the dorsal PCs of the hindgut using *en-*Gal4. Similarly as already reported [14], [15], [16], [17] overexpressed Crb becomes ectopically localised and recruits other apical proteins (e. g. *D*Par6) to ectopic sites in epithelial cells of the large intestine (Fig. 6). The epithelium is disorganised, making any quantification of microvilli impossible.

## Discussion

In most *Drosophila* tissues studied so far, apical localisation of Crb depends on Sdt, mediated by direct interaction between the C-terminus of Crb and the PDZ-domain of Sdt [23], [24], [39], [61]. This interaction is conserved in vertebrates [62], [63]. Here we show that neither the C-terminal PDZ-domain binding motif nor an intact FERM-domain binding motif of the cytoplasmic domain of Crb is required for proper localisation of Crb in the BCs of the hindgut. A Crb protein lacking the C-terminal ERLI motif, as in *foscrb_Y10A,ΔERLI_;crb^GX24^* [33] or in *crb^8F105^* [32] still accumulates apically in the BCs. Unlike cells of the Malpighian tubules, which show apical localisation of the Crb^8F105^ protein at early (stage 11), but not at late stages (stage 16) [64], the mutant protein remains apically in the BCs even at late stages. This suggests that stabilisation of Crb does not require the interaction with a PDZ-domain containing protein in these cells, thus also excluding *D*Par6. Since all (verified and predicted) Sdt isoforms contain a PDZ domain (Flybase) and all *sdt* alleles described so far are protein null in the embryo, as revealed by using an antibody directed against the PDZ domain [39], we find it rather unlikely that an unknown isoform of Sdt is involved in the stabilisation of Crb in the BCs. Furthermore, an intact FERM-domain binding domain in Crb is not required for apical localisation of Crb, as revealed by proper apical enrichment of Crb in the BCs of *foscrb_Y10A,ΔERLI_;crb^Gx24^* embryos. We assume that the Y10A exchange abolishes the FERM-binding function. This conclusion is based on data showing that a tyrosine residue at position 10 in the FERM-domain binding motif of the adhesion molecule ICAM-2 is part of the peptide that participates in intimate interactions with the FERM-domain of radixin. Exchange of this tyrosine by alanine caused a 16 fold reduction in the binding affinity to the FERM-domain of radixin [65].

So far, we can only speculate whether the 14 amino acids still present in the truncated cytoplasmic domain of the Crb protein encoded by *crb^8F105^* [32] contain a yet undefined apical targeting and/or retention sequence. If so, this sequence is acting in a cell-type specific way, since the mutant Crb protein, which lacks both the PDZ- and FERM- domain binding motif is still apically localised in BCs, but not in PCs. All predicted *Drosophila* Crb isoforms contain the same cytoplasmic tail composed of 37 amino acids, but we cannot exclude the possibility that an alternative form is expressed in BCs. The human *CRB3* gene encodes two isoforms due to alternative splicing, one of which, CRB-CLPI, contains an alternative C-terminus that lacks the conserved –ERLI motif. This isoform is specifically localised in cilia of fully differentiated Madin-Darbine canine kidney (MDCK) cells, but not in newly polarised cells still lacking cilia [66].

Various mechanisms have been described that are involved in the stabilisation/retention of proteins on the apical surface. For example, the stability of the apical Cystic Fibrosis Transmembrane Conductance Regulator (CFTR), a cyclic AMP-regulated chloride channel with an important role in the control of the volume of the lung airway surface liquid, can be regulated by various interactions mediated by its cytoplasmic domains. Beside the interaction of its C-terminus with the PDZ domain of EBP50 (ERM-binding phosphoprotein 50), the stability of CFTR was shown to depend on interactions of a hydrophobic motif with the intermediate filament protein keratin 18, or by interaction of an N-terminal sequence with the actin-binding protein filamin-A [67], [68], [69], [70]. An alternative way to stabilise Crb apically could be via homophilic cis-interactions mediated by the extracellular domains of Crb proteins, which is still present in the protein encoded by the mutant *crb^8F105^* allele. Homophilic interactions have recently been suggested as a mechanism for Crb stabilisation in the embryonic epidermis and the follicle epithelium of *Drosophila* [71], [72] and in the zebrafish retina, where they mediate the formation of the cone mosaic [73]. Alternatively, another, yet unknown protein specifically expressed in the BCs could stabilise Crb by heterophilic interactions of the extracellular domains.

The BCs of the large intestine not only differ from the PCs by a different mechanism for Crb stabilisation, but also by a much higher level of Crb protein on the apical surface, which is associated with a higher transcript level [28]. As shown here, the high level of Crb expression has an influence on the length of the microvilli in these cells, but not on the formation of the microvilli per se. This is in line with results obtained from overexpression in other epithelial cells, which can induce enlarged or ectopic apical surfaces [14], [17]. Experimentally induced overexpression of Crb in dorsal PCs results in ectopic apical proteins. Due to the disorganisation of the epithelium microvilli length could not be measured. An interesting speculation to explain Crb accumulation in the BCs relies on a reverse scenario, in which microvilli are required for apical retention of membrane proteins. This mechanism has been recently derived from studies in mice lacking the three actin-bundling proteins villin, espin and plastin-1. Enterocytes of triple knock-out mice do form microvilli, which lack, however, the typical actin filament bundles. Strikingly, apical transmembrane proteins and enzymes are poorly retained [74]. Assuming a similar mechanism in the *Drosophila* hindgut, the stronger accumulation of Crb in BCs could be a consequence of longer microvilli. Similar as in *crb*, microvilli length of BCs is also reduced in embryos mutant for *slit* or *robo*, but increased in *robo2* and *robo/robo2* double mutants. However, no difference in Crb staining was found in the BCs of these mutants compared to that of wild-type [60]. This indicates that there is no obvious dependence of Crb abundance on the length of the microvilli of BCs.

Alternatively, a different physiological state of BCs and PCs could be responsible for the different behaviour of Crb. Epithelial cells of the proximal renal tubule of acidotic rats, for example, adapted to this change by altering the protein composition in the microvilli and the apical cortex. The change included transmembrane proteins such as transporters, but also scaffolding proteins or proteins involved in trafficking [75]. Finally, microvilli in BCs could define a distinct lipid microdomain responsible for plasma membrane domain-specific retention. For example, segregation of different raft-associated gangliosides into microvilli or the smooth portion of the apical membrane of MDCK cells correlate with the differential segregation of the pentaspan protein prominin-1 in microvilli [76], [77]. Interestingly, the single-span transmembrane protein Stranded-at-Second is reduced on the apical microvilli of BCs in comparison to its level in the PCs (see Fig. 3), supporting the idea that the expanded apical membrane of the BCs may regulate differential retention of only a subset of proteins. Whether any of the discussed mechanisms is used by the BCs to enrich Crb on the apical surface and which function the BCs have in the hindgut requires further investigation.
